# The moose throat bot fly *Cephenemyia ulrichii* larvae not found developing in roe deer

**DOI:** 10.1186/s13028-022-00663-w

**Published:** 2022-12-30

**Authors:** Petra Heikkinen, Marja Isomursu, Antti Oksanen

**Affiliations:** Animal Health Diagnostic Unit (FINPAR), Finnish Food Authority, Oulu, Finland

**Dear editor,**

In 2008, we published a report on *Cephenemyia ulrichii*, the moose (*Alces alces*) throat bot in roe deer (*Capreolus capreolus*) [1]. The identification was based on morphology of two 3rd instar larvae of around 50 seen in a spring hunted roe deer buck shot in Kirkkonummi, Finland. The morphological characteristics utilized in species identification as *C. ulrichii* were especially spines irregularly placed on the anterior dorsal side, while those of *C. stimulator* and *C. trompe* are in regular rows similar to those on the ventral surfaces. The only deviation we could find from the previously published characteristics [2] was the smaller size, about 26–27 mm long, while those of *C. ulrichii* typically reach a length of 40 mm.

Because there have been a number of reports of throat bots from roe deer in Finland in the last few years, and some of them have genetically been positively identified as *C. stimulator* (unpublished), we took the remaining (third) larva from the original sample and performed limited morphological analysis, which showed the morphology was consistent with the other two larvae previously identified as *C. ulrichii* with irregularly located spines on the anterior dorsal side (Fig. 1). In addition, we performed PCR and subsequent sequencing of the CO1 gene, which unequivocally proved the larva to be *C. stimulator*, not *C. ulrichii*.

**Fig. 1:**
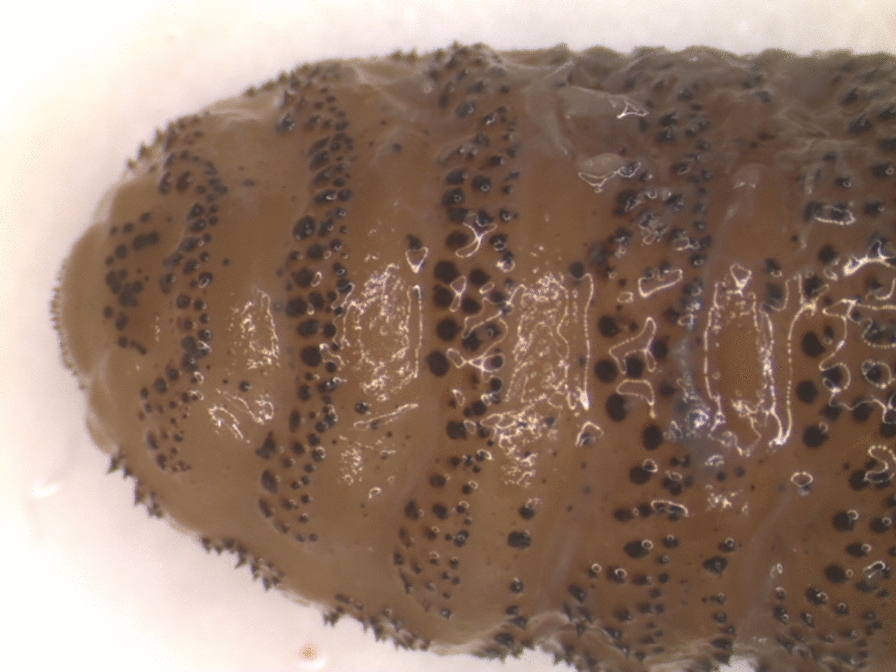
Dorsal anterior view of the *Cephenemyia stimulator* larva collected 2007, analyzed 2022

The discrepancy between morphological and genetic diagnoses shows that morphological characteristics of *Cephenemyia* spp. 3rd instar larvae are not unambiguous. Fortunately, genetic analyses are easily performed now, unlike just 15 years ago.

To our knowledge, no further reports on *C. ulrichii* in roe deer have been published since our initial, hereby cancelled, one in 2008. We then concluded: “Generally, all *Cephenemyia* species are very host specific and thereby also well adapted to their hosts”. This is even truer than we then had reason to believe.

*Cephenemyia stimulator* has since been documented in Skåne, Southern Sweden, in 2012 [3].

## Data Availability

The data that support the findings of this study are available from the corresponding author upon reasonable request.
